# Unraveling the subtleties of β-(1→3)-glucan phosphorylase specificity in the GH94, GH149, and GH161 glycoside hydrolase families

**DOI:** 10.1074/jbc.RA119.007712

**Published:** 2019-02-28

**Authors:** Sakonwan Kuhaudomlarp, Giulia Pergolizzi, Nicola J. Patron, Bernard Henrissat, Robert A. Field

**Affiliations:** From the ‡ Department of Biological Chemistry, John Innes Centre, Norwich Research Park, Norwich NR4 7UH, United Kingdom,; the § Earlham Institute, Norwich Research Park, Norwich NR4 7UZ, United Kingdom,; ¶ Architecture et Fonction des Macromolécules Biologiques, Aix-Marseille University, 163 Avenue de Luminy, 13288 Marseille, France,; ‖ CNRS, UMR 7257, 163 Avenue de Luminy, 13288 Marseille, France, and; the ** Department of Biological Sciences, King Abdulaziz University, Jeddah 23218, Saudi Arabia

**Keywords:** Gram-positive bacteria, carbohydrate metabolism, glycoside hydrolase, glycobiology, phosphorylase, β-1,3-glucan, carbohydrate utilization loci, enzyme evolution, heterokont, orthologs

## Abstract

Glycoside phosphorylases (GPs) catalyze the phosphorolysis of glycans into the corresponding sugar 1-phosphates and shortened glycan chains. Given the diversity of natural β-(1→3)-glucans and their wide range of biotechnological applications, the identification of enzymatic tools that can act on β-(1→3)-glucooligosaccharides is an attractive area of research. GP activities acting on β-(1→3)-glucooligosaccharides have been described in bacteria, the photosynthetic excavate *Euglena gracilis*, and the heterokont *Ochromonas* spp. Previously, we characterized β-(1→3)-glucan GPs from bacteria and *E. gracilis*, leading to their classification in glycoside hydrolase family GH149. Here, we characterized GPs from Gram-positive bacteria and heterokont algae acting on β-(1→3)-glucooligosaccharides. We identified a phosphorylase sequence from *Ochromonas* spp. (OcP1) together with its orthologs from other species, leading us to propose the establishment of a new GH family, designated GH161. To establish the activity of GH161 members, we recombinantly expressed a bacterial *GH161* gene sequence (PapP) from the Gram-positive bacterium *Paenibacillus polymyxa* ATCC 842 in *Escherichia coli*. We found that PapP acts on β-(1→3)-glucooligosaccharide acceptors with a degree of polymerization (DP) ≥ 2. This activity was distinct from that of characterized GH149 β-(1→3)-glucan phosphorylases, which operate on acceptors with DP ≥ 1. We also found that bacterial *GH161* genes co-localize with genes encoding β-glucosidases and ATP-binding cassette transporters, highlighting a probable involvement of GH161 enzymes in carbohydrate degradation. Importantly, in some species, *GH161* and *GH94* genes were present in tandem, providing evidence that GPs from different CAZy families may work sequentially to degrade oligosaccharides.

## Introduction

Diverse β-(1→3)-glucan structures can be found in bacteria (1), fungi (2), plants (3), and algae (4–6), from which they are extracted for use in a wide range of biotechnological applications, including as ingredients in agricultural, food, cosmetic, and therapeutic products (7). For example, β-(1→3)-glucans have been used as food stabilizers, and there is much interest in their application as functional dietary fiber supplements (8) and as immunomodulatory agents (9, 10). Anti-tumor properties have also been reported, often in correlation with immunomodulatory effects (11). These diverse applications prompt the search for efficient, low-cost production of β-(1→3)-glucan in defined form (*e.g.* specific degrees of polymerization).

**Figure 1.**
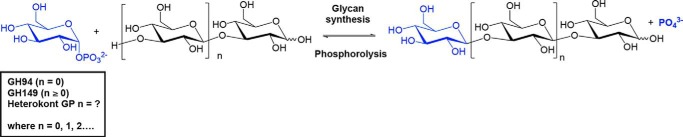
**Activities of β-(1→3)-glucan phosphorylases.** The chain length specificity of the heterokont enzyme (classified as GH161 in this work) was unknown.

Glycoside phosphorylases (GPs)^3^ are a group of enzymes catalyzing reversible phosphorolysis of glycans into the corresponding sugar 1-phosphates and shortened glycan chains (12, 13). The reversibility of the reaction also enables production of lengthened glycans from the sugar 1-phosphate donor and glycan acceptor of choice. Sugar 1-phosphate donors for GPs are relatively cheap and accessible compared with the nucleotide sugars required for glycosyltransferases, therefore making GPs attractive as biocatalysts for β-(1→3)-glucan production. GPs acting on β-d-glucopyranosyl-(1→3)-d-glucopyranose (laminaribiose) (Fig. 1) have been described previously from the bacteria, *Paenibacillus* sp. YM-1 (PsLBP) (14) and *Acholeplasma laidlawii* PG-8A (15). Identification of their gene sequences enabled their classification into glycoside hydrolase (GH) family 94 (16, 17). GPs acting on longer β-(1→3)-d-gluco-oligosaccharides have been described in eukaryotic microalga from different lineages: euglenozoans (*Euglena gracilis*) (18–20) and heterokonts (*Ochromonas danica* (21) and *Ochromonas malhemensis* (22)). Previously, we identified genes encoding a β-(1→3)-glucan phosphorylase from *E. gracilis* (EgP1) and a bacterial ortholog thereof from a metagenomic source (Pro_7066) (23). Functional assays of recombinant EgP1 and Pro_7066 proteins confirmed their function as β-(1→3)-glucan phosphorylases. These enzymes and their orthologs constituted a new family of GH-like GPs (GH149). Structural studies (24)^4^ and multiple-sequence alignments confirmed the conservation of key amino acid residues involved in catalysis and validated the placement of both families within the same GH clan (GH-Q) (24). However, genes that relate to the β-(1→3)-d-glucan phosphorylase activity from heterokonts have yet to be described. Here, β-(1→3)-d-glucan phosphorylases are likely involved in the metabolism of chrysolaminarin, a soluble β-(1→3)-glucan with a limited degree of β-(1→6)-branches (25, 26), that accumulates within the vacuole of photosynthetic heterokonts (27).

Availability of genomes and transcriptomes of two model heterokont species, *Phaeodactylum tricornutum* and *Thalassiosira pseudonana*, has enabled the study of chrysolaminarin metabolism. Genome analysis of *P. tricornutum* led to the identification of a UDP-glucose pyrophosphorylase, a β-(1→3)-d-glucan synthase, and enzymes generating β-(1→6) branches (28, 29). Likewise, analysis of the *T. pseudonana* genome identified several sequence candidates involved in chrysolaminarin metabolism (30). However, these studies did not identify GPs, likely due to the lack of sequence information corresponding to phosphorylase activity. Therefore, the identification of candidate sequences is a key step in the investigation of heterokont β-(1→3)-d-glucan phosphorylases.

In continuation of our work on the identification and characterization of new β-(1→3)-d-glucan phosphorylases (12, 23, 24, 31), a heterokont phosphorylase sequence from *Ochromonas* spp. (OcP1) was identified together with bacterial orthologs, which has subsequently led to the establishment of a new GH family, designated GH161. In contrast to GH149, for which the majority of sequences are from Gram-negative bacteria and Euglenophyceae, the majority of GH161 sequences were identified from the genomes of Gram-positive bacteria and heterokonts. Despite repeated attempts, expression of recombinant OcP1 protein in *Escherichia coli* was unsuccessful. To establish the activity of GH161 family members, a bacterial *GH161* gene sequence (*PapP*) from the Gram-positive bacterium *Paenibacillus polymyxa* ATCC 842 was cloned and expressed in *E. coli*. Biochemical characterization of recombinant PapP protein showed that the enzyme operates on β-(1→3)-gluco-oligosaccharide acceptors with a degree of polymerization (DP) ≥ 2 and that it cannot use monosaccharide glucose (Glc) as an acceptor substrate. The acceptor length specificity of PapP is distinct from that of the characterized GH149 enzymes, which can act on Glc (Fig. 1). Bacterial *GH161* genes were found in close proximity to genes encoding β-glucosidases (GH1, GH3, or GH30) and ATP-binding cassette (ABC) transporters, highlighting a probable involvement of these gene clusters in carbohydrate degradation. Significantly, some *GH161* genes were found located adjacent to genes in the *GH94* family, suggesting that GPs from different CAZy families may work in concert to enable sequential degradation of oligosaccharides.

## Results

### Analysis of the heterokont genomes reveals candidate genes encoding β-(1→3)-d-glucan phosphorylases

*Ochromonas* β-(1→3)-glucan phosphorylases were expected to share residual sequence similarity to genes encoding these enzymes in the GH94 and GH149 families. To investigate this hypothesis, the translated transcriptome of *Ochromonas* sp. BG-1 from the Marine Microbial Eukaryote Transcriptome (MMET) database was interrogated using BLASTP with GH94 (21 sequences) and GH149 EgP1 sequences as queries. Three sequences were recovered (CAMPEP_0173148212, CAMPEP_0173133844, and CAMPEP_0173155066), with 21% identity to a GH94 laminaribiose phosphorylase (LBP) sequence from *Halorhadus tiamatea* and 24% to EgP1 sequence (Table 1). These three sequences were found to be 99% identical, and therefore CAMPEP_0173155066 was taken forward as a representative, designated OcP1 (see supporting information for the full sequence).

**Table 1:** **BLASTP analysis of *Ochromonas* sp. transcriptomic sequences using 21 sequences of GH94 family and EgP1 of GH149 as queries**

Query	Subject	Hit ID	Query coverage	*E*-value	Identity
			%		%
WP_020936056.1|LBP GH94| (*Halorhabdus tiamatea*)	*Ochromonas* sp. strain BG-1 transcriptome (MMETSP1105)	CAMPEP_0173148212	65	3e−06	21
		CAMPEP_0173133844	65	8e−06	21
		CAMPEP_0173155066 (OcP1)	65	8e−06	21
EgP1|GH149 (*Euglena gracilis*)	*Ochromonas* sp. strain BG-1 transcriptome (MMETSP1105)	CAMPEP_0173148212	31	3e−08	24
		CAMPEP_0173133844	31	3e−08	24
		CAMPEP_0173155066 (OcP1)	31	3e−08	24

**Figure 2.**
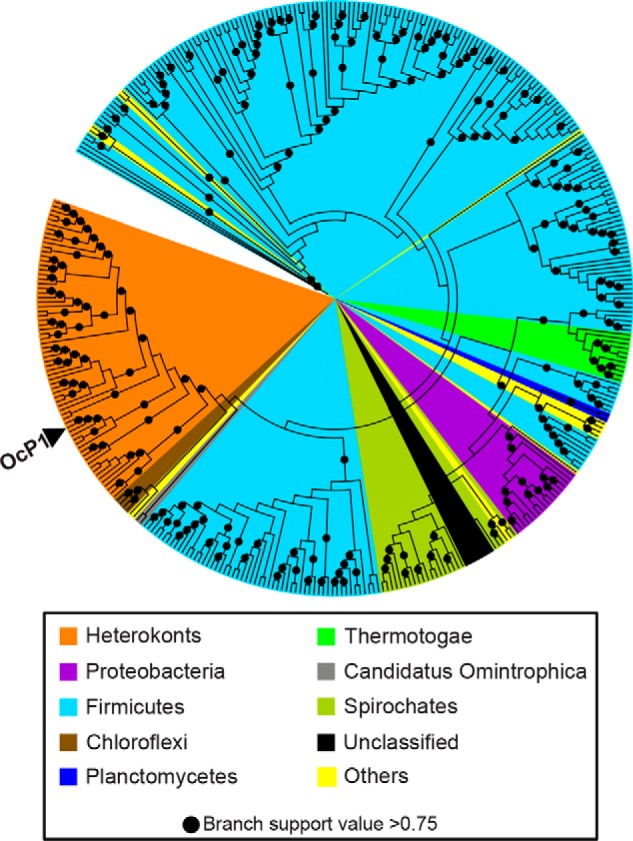
**Phylogenetic analysis of OcP1 and its orthologs.** The position of the OcP1 sequence is indicated by an *arrowhead*.

Interrogation of the NCBI nonredundant protein database and the MMET database using PSI-BLAST with OcP1 as a query uncovered several hundred orthologs (File S1). Of these, 69 sequences (with 32–58% sequence identity to OcP1; File S2) originated from eukaryotes in the phyla Bacillariophyta, Ochrophyta, Haptophyta, and Miozoa, all of which belong to infrakingdom Heterokonta (collectively termed heterokonts) (Fig. 2, *orange*). One sequence, GenBank^TM^ accession number BAU78234.1 (identity = 58%, *E*-value = 0), was identified from *Ochromonas danica*. The remaining 332 sequences identified that share 31–41% sequence identity to OcP1 (File S2) were from Gram-positive bacteria in the phylum Firmicutes (Fig. 2, *cyan*; 229 sequences), 85 of which were from *Paenibacillus* spp.

**Figure 3.**
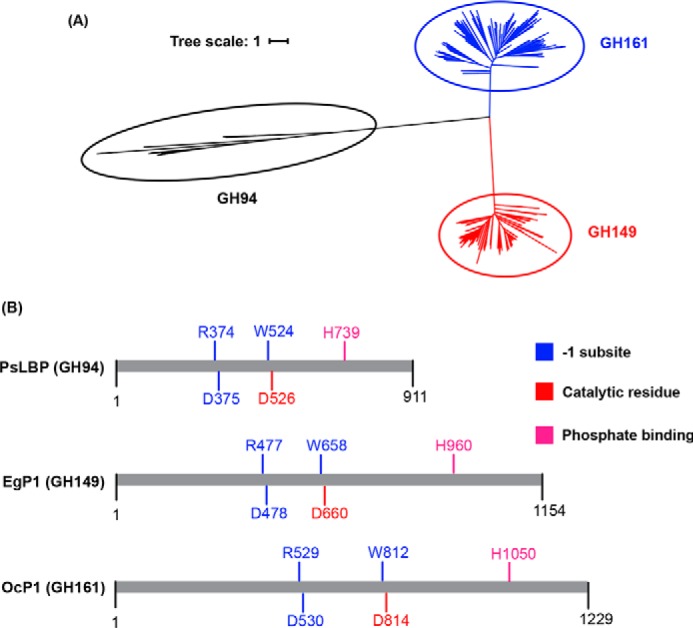
**Relationship between GH94, GH149, and GH161.** *A*, an unrooted tree representing the phylogenetic relationship between GH94 queries (*black branches*), GH149 (*red branches*), and GH161 (*blue branches*). *B*, summary of sequence alignment of GH94 (PsLBP), GH149 (EgP1), and GH161 (OcP1).

To establish the relationship between OcP1 orthologs and GH families containing β-(1→3)-d-glucan phosphorylases (GH94 and GH149), phylogenetic relationships were reconstructed using amino acid sequences. Maximum likelihood analyses indicated that the OcP1 orthologs form a distinct clade that is alienated from GH94 and GH149 (Fig. 3*A*, *blue*); thus, OcP1 and its orthologs constitute a new family, designated GH161. Despite their low overall sequence similarity (∼20% across the three families), multiple-sequence alignment of amino acid sequences revealed conservation of aspartate catalytic residues, residues located in the −1 (donor substrate) subsite, as well as a histidine residue involved in phosphate recognition previously identified in GH94 and GH149 (Fig. 3*B*). Together, these data support the placement of the GH161 family into the GH-Q clan, together with GH149 and GH94.

Notably, sequences in the GH161 family were identified in the genomes of heterokonts for which other chrysolaminarin metabolic enzymes have previously been identified (28–30). By analysis of the heterokont genomes, we were able to analyze genomic sequences and found that all genes contain at least one intron (Table S1). Introns were also identified previously in other β-(1→3)-glucan metabolic enzymes, such as laminarin synthase from *P. tricornutum* (32), and exclude the possibility that these sequences derive from bacterial contamination.

### Biochemical characterization of recombinant GH161 proteins reveal phosphorylase activity on β-(1→3)-glucan chains

Although many conditions were trialed, we were unable to express recombinant OcP1 protein in *E. coli*. To characterize the bacterial activity of enzymes in the GH161 family, candidates from *Paenibacillus* spp. were selected because several studies report that *Paenibacillus* carbohydrate-active enzymes (CAZymes) can act on β-(1→3)-glucan and mixed linkage β-(1→3;1→4)-glucan (*e.g.* identification of a β-(1→3)-*endo*-glucanase (LamA) in *Paenibacillus* sp. CCRC 17245 able to digest β-(1→3;1→4)-glucan (33)). Also, a laminarin-degrading β-glucanase (BglA_1_) and a β-(1→3;1→4)-*endo*-glucanase (BglA_2)_ have been identified from *Paenibacillus* sp. JDR-2 (34), along with β-(1→3;1→4)-*endo*-glucanase from *Paenibacillus* sp. F-40 (35). Second, an LBP from *Paenibacillus* sp. YM-1 (PsLBP) in the GH94 family has been described previously (14). Therefore, characterization would enable comparison between the GH94, GH149, and GH161 β-(1→3)-d-glucan phosphorylases and perhaps provide further insights into their newly discovered activity.

A GH161 sequence from *Paenibacillus polymyxa* ATCC 842 (GenBank^TM^ accession number WP_019688419.1, 37% identity to OcP1), designated *PapP*, was chosen as a target for recombinant protein expression and *in vitro* biochemical characterization. The coding sequence (gene locus: PPT_RS0121460) was amplified and cloned into a pOPINF vector (36), which installs a fused N-terminal His_6_ tag, and expressed in *E. coli* (Rosetta PLysS). The recombinant PapP protein was purified by immobilized metal affinity chromatography (IMAC) (Fig. S1, *A* and *B*) and subsequently by gel filtration (Fig. S1*C*). Protein with an approximate molecular mass of 120 kDa, based on SDS-PAGE analysis, was recovered (Fig. S1*D*). Gel filtration analysis of PapP against protein standards revealed that PapP formed a dimer in solution under nondenaturing conditions, with an estimated molecular mass of 241 kDa. A dimeric form of PapP is similar to that described for PsLBP, EgP1, and Pro_7066 (Table S2).

To investigate function, the recombinant PapP was assayed *in vitro* in a glycan synthetic reaction using Glc and Glc1P as acceptor and donor substrates, respectively. The reaction mixture was analyzed by thin layer chromatography (TLC), which showed that there was no detectable activity on these substrates (Fig. S2*A*). Similarly, phosphorolysis assays were performed in the presence of Glc-Glc disaccharides with varying glycosidic linkages as substrates, again showing no turnover (Fig. S2*B*). These initial experiments suggested that PapP may prefer substrates with a higher DP.

**Figure 4.**
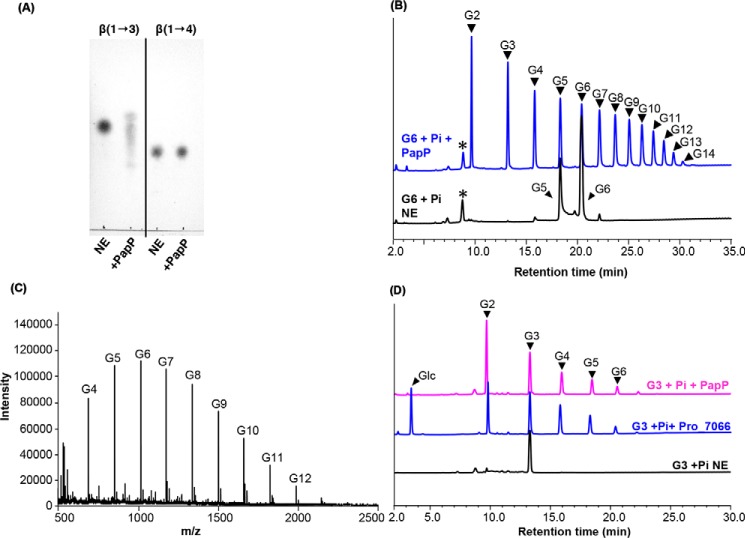
**Characterization of PapP and comparison of PapP substrate chain length preference with that of GH149 enzyme.** *A*, TLC analysis of the phosphorolysis reaction carried out by PapP in the presence of 20 mm laminaritriose (β-(1→3)-linkage) or cellotriose (β-(1→4)-linkage). *B*, HPAEC-PAD analysis of the phosphorolysis reaction of 20 mm G6 carried out by PapP in the presence of 10 mm P_i_. G5 was detected as a contaminant in the starting material. *C*, MALDI-TOF analysis of the G6 + P_i_ + PapP reaction in *B*. G2–*G14*, DP of the β-(1→3)-gluco-oligosaccharide products. *NE*, no enzyme control. All assays were performed in 100 mm HEPES, pH 7.0, at 30 °C for 1 h. *, unknown peaks. *D*, comparison between the phosphorolysis carried out by either PapP (magenta) or Pro_7066 from GH149 family (*blue*) in the presence of 20 mm G3 and 10 mm P_i_ as substrates.

The enzyme was assayed in a phosphorolysis reaction in the presence of a trisaccharide (either β-(1→3)- (laminaritriose, G3) or β-(1→4)-oligosaccharides (cellotriose)) and P_i_ as substrates, which showed that PapP could phosphorolyse laminaritriose but not cellotriose. This confirms that PapP prefers β-(1→3)-glycosidic linkages (Fig. 4*A*), which was substantiated in the phosphorolysis direction with laminarihexaose (G6) and P_i_, which showed that G6 was broken down by PapP into shorter oligosaccharides (G2–G5). However, in contrast to the characterized GH94 and GH149 enzymes, PapP could not phosphorolyse laminaribiose (G2) to Glc (Fig. 4*B*). Because the phosphorolysis is reversible, peaks corresponding to oligosaccharide products longer than the G6 substrate were also detected (G7–G14; Fig. 4, *B* and *C*, and Table S3). Previously, two GH149 enzymes (EgP1 and Pro_7066) were confirmed as β-(1→3)-d-glucan phosphorylases that can carry out the phosphorolysis of β-(1→3)-oligosaccharides to liberate Glc, representing a different substrate preference from the GH161 enzyme PapP. To emphasize the difference in substrate chain length preferences between the GH149 and GH161 enzymes, assays were carried out in the presence of G3 and P_i_ as substrates using either PapP or Pro_7066 as an enzyme catalyst. The reactions were analyzed by high-performance anion-exchange chromatography with pulsed amperometric detection (HPAEC-PAD), which showed that Pro_7066 was capable of degrading G3 to Glc, whereas PapP only degraded G3 to G2 (Fig. 4*D*). Altogether, these results strongly indicate that PapP is a β-(1→3)-d-glucan phosphorylase that can phosphorolyse linear oligosaccharide chains with DP ≥ 3, distinguishing this enzyme as distant from its GH94 and GH149 relatives.

To further probe the acceptor chain length specificity of PapP, kinetic analyses were performed in the glycan synthetic direction in the presence of β-(1→3)-gluco-oligosaccharides (G2–G6) as acceptors and Glc1P as a donor. PapP showed similar catalytic efficiency (*k*_cat_/*K_m_*) toward all acceptor chain lengths (G2–G6) when 10 mm Glc1P was used as a donor, but no detectable activity on Glc. In contrast, EgP1 and Pro_7066 from GH149 were at least as proficient with Glc as an acceptor as with longer β-(1→3)-gluco-oligosaccharides (24) (Table 2).

**Table 2:** **Kinetic parameters of the glycan synthesis reaction catalyzed by PapP when 10 mm Glc1P was used as a donor** Kinetic parameters for GH149 enzymes (EgP1 and Pro_7066) reported previously are presented for comparison (24). NA, no activity.

Acceptor	PapP			EgP1			Pro_7066		
	*k*_cat_	*K_m_*	*k*_cat_/*K_m_*	*k*_cat_	*K_m_*	*k*_cat_/*K_m_*	*k*_cat_ (s^−1^)	*K_m_*	*k*_cat_/*K_m_*
	*s*^−*1*^	*mm*	*s*^−*1*^ *mm*^−*1*^	*s*^−*1*^	*mm*	*s*^−*1*^ *mm*^−*1*^	*s*^−*1*^	*mm*	*s*^−*1*^ *mm*^−*1*^
Glc	NA	NA	NA	1.10 ± 0.03	0.56 ± 0.06	1.99	1.66 ± 0.04	0.29 ± 0.03	5.79
G2	31.6 ± 1.0	1.58 ± 0.17	20.0	1.08 ± 0.03	0.67 ± 0.08	1.62	1.54 ± 0.01	0.25 ± 0.02	6.03
G3	33.3 ± 1.6	1.05 ± 0.19	31.6	1.12 ± 0.02	1.26 ± 0.09	0.89	1.53 ± 0.02	0.37 ± 0.03	4.16
G4	33.3 ± 1.7	1.82 ± 0.29	18.3	1.12 ± 0.03	1.41 ± 0.13	0.79	1.39 ± 0.01	0.36 ± 0.02	3.89
G5	27.4 ± 0.7	1.61 ± 0.13	17.0	1.13 ± 0.03	2.29 ± 0.19	0.50	1.27 ± 0.01	0.32 ± 0.02	4.04
G6	30.0 ± 0.6	2.31 ± 0.56	13.0	1.10 ± 0.03	2.88 ± 0.23	0.38	1.18 ± 0.04	0.26 ± 0.04	4.62

**Figure 5.**
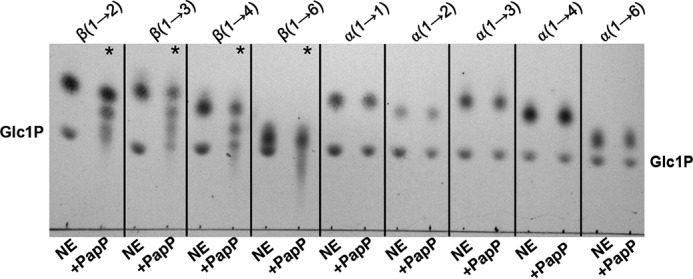
**Glycan synthetic reactions carried out by PapP in the presence of β-linked disaccharide acceptors and Glc1P.** TLC analysis of the glycan synthesis in the presence of Glc-Glc disaccharides (linkages as indicated at the *top*). *, detectable extension of the acceptors carried out by PapP.

PapP required a substrate with a minimum DP of 3 for phosphorolysis, implying that the enzyme must be able to use a disaccharide (DP = 2) for the glycan synthetic reaction. Therefore, assays were performed in the presence of Glc-Glc disaccharides with varying glycosidic linkages as acceptors and Glc1P as a donor. Interestingly, PapP could use other β-linked disaccharides as acceptors, regardless of the linkage regioselectivity ((1→2), (1→3), (1→4), or (1→6)), whereas there was no detectable activity on isomeric α-linked Glc-Glc disaccharides (Fig. 5 and Fig. S3). The relaxed acceptor regioselectivity observed in PapP suggested that this function might also be a property of GH149 enzymes. To investigate this hypothesis, Pro_7066 was assayed in the glycan synthetic reactions in the presence of Glc-Glc disaccharides with varying glycosidic linkages, which also showed glycan synthetic activity toward isomeric β-linked acceptors in the same manner as for PapP (Figs. S4 and S5).

**Figure 6.**
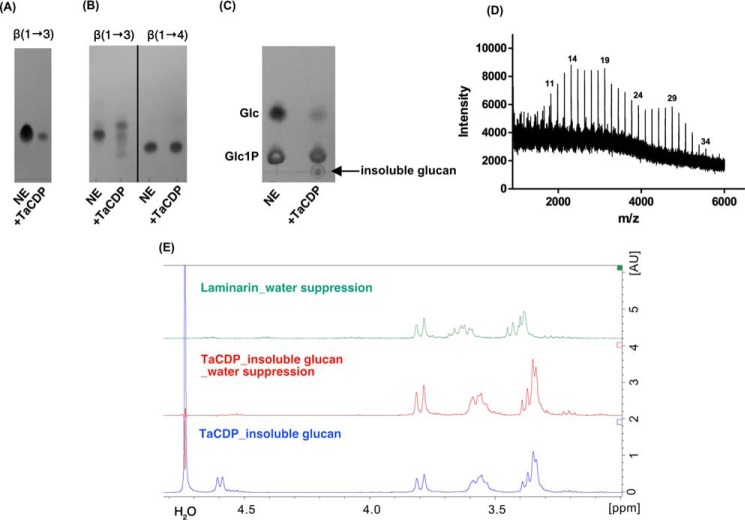
**Characterization of TaCDP.** *A*, TLC analysis of the phosphorolysis reaction carried out by TaCDP in the presence of 20 mm laminaribiose and 10 mm P_i_; *B*, TLC analysis of the phosphorolysis reaction carried out by TaCDP in the presence of 20 mm laminaritriose [β-(1→3)] or cellotriose [β-(1→4)] and 10 mm P_i_. All enzymatic reactions in *A* and *B* were performed at 37 °C for 12 h in 100 mm HEPES, pH 7.2. *NE*, no enzyme control. *C*, glycan synthetic reaction in the presence of Glc (10 mm) and Glc1P (50 mm) in 250 mm HEPES, pH 7.2, incubated at 37 °C for 4 days. *D*, MALDI-TOF analysis of the insoluble glucan product from *C*. The corresponding DPs of the glucan product are indicated at the *top* of the peaks. *E*, ^1^H NMR analysis (400 MHz, 25 °C) of the insoluble product from *C*. The insoluble glucan was washed three times with D_2_O, freeze-dried, and redissolved in 1 m NaOD prior to the analysis. ^1^H NMR analysis of the insoluble product (*blue*) shows a doublet centered at 4.6 ppm, indicating the signal of the axial H-1 protons of the internal β-(1→3)-linkages. The doublet disappeared on ^1^H NMR of the same sample under water suppression (*red*). The ^1^H NMR spectrum of laminarin (β-(1→3)-glucan) standard recorded under water suppression is included for comparison (*green*).

During bioinformatics analyses of GH161 sequences in the NCBI database, we came across sequence from the Gram-negative thermophilic bacterium *Thermosipho africanus* TCF52B (TaCDP, GenBank^TM^ number ACJ76363.1) that had recently been reported as a cellodextrin (β-(1→4)-glucan) phosphorylase, operating on Glc and cello-oligosaccharides (37). This was at odds with our expectations and warranted further investigation, given that TaCDP phosphorolytic activity on β-(1→3)-gluco-oligosaccharide substrates had not been assessed and the glucan products for β-(1→4)-glucan extension had not been subjected to linkage analysis. To confirm the substrate specificity of TaCDP enzyme, the TaCDP gene sequence (GenBank^TM^ number CP001185.1, locus 1948177–1951236) was codon-optimized for *E. coli* expression, and the synthetic gene was amplified by PCR and subsequently cloned into a pOPINF vector. The gene was expressed in *E. coli* BL21 (DE3), and the resulting recombinant protein was purified by IMAC. To establish the substrate specificity of TaCDP, we assayed the enzyme in the phosphorolytic reaction against laminaribiose, cellotriose, and laminaritriose in the same manner as described for PapP. This showed that TaCDP was active exclusively on laminaritriose, with no detectable activity on either laminaribiose or cellotriose (Fig. 6, *A* and *B*). The enzyme also showed weak activity on Glc as an acceptor in the presence of Glc1P, producing an insoluble product after 4 days of incubation at 37 °C (Fig. 6, *C* and *D*). MALDI-TOF MS analysis of the insoluble product (Fig. 6*D*) showed the formation of oligosaccharides of DP to at least 34, which is far in excess of the product range produced by an authentic CDP, which typically precipitates at DP ∼9. In addition, upon redissolving the TaCDP-derived insoluble polymer in 1 m NaOD, ^1^H NMR analysis showed a doublet centered at 4.6 ppm, representing the signal of the axial H-1 protons of the internal β-(1→3)-linkages, confirming that the product is a β-(1→3)-linked gluco-oligosaccharide and not a cellodextrin (Fig. 6*E*).

### The identities of genes in bacterial gene clusters containing GH161 genes suggest specialized roles in carbohydrate degradation

**Figure 7.**
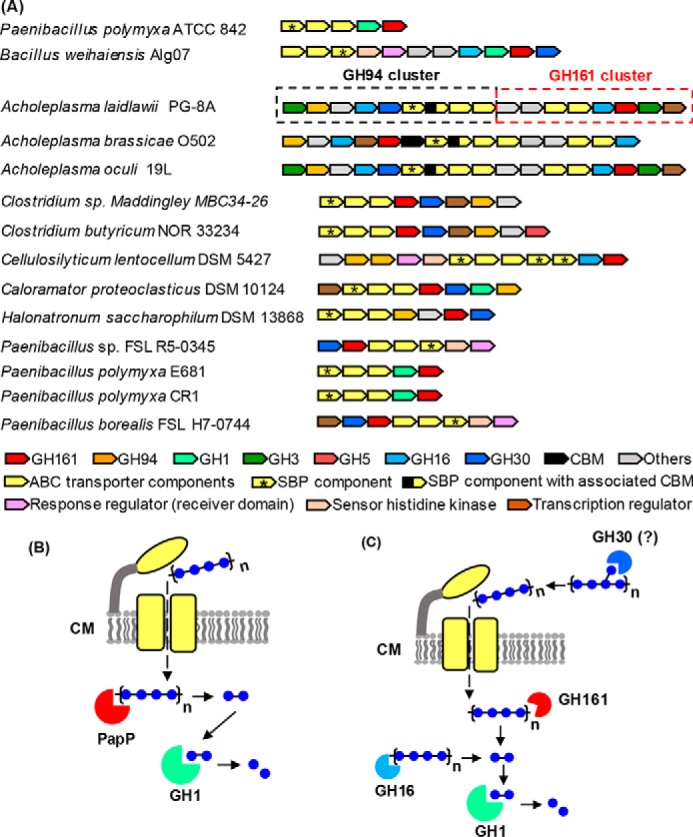
**Genetic clusters containing genes encoding GH161 and other glucosidases.** The gene cluster containing the previously characterized GH94 LBP from *A. laidlawii* PG-8A is indicated by a *black-dotted box* (15), whereas the adjacent GH161 gene cluster is indicated by a *red-dotted box*. SBP components of the ABC transport system are indicated by *asterisks. B* and *C*, predicted roles of the enzymes encoded by the gene clusters in *A*. ABC transporters are *colored* in *yellow* and located in the cell membrane (*CM*). The enzymes are *colored* according to the *colors* of the corresponding genes presented in the gene clusters. The subcellular localization prediction of GHs and transporters was performed by PSORTb version 3.0.2 (http://psort.org/psortb/index.html)^5^ using default settings for Gram-positive bacteria.

**Figure 8.**
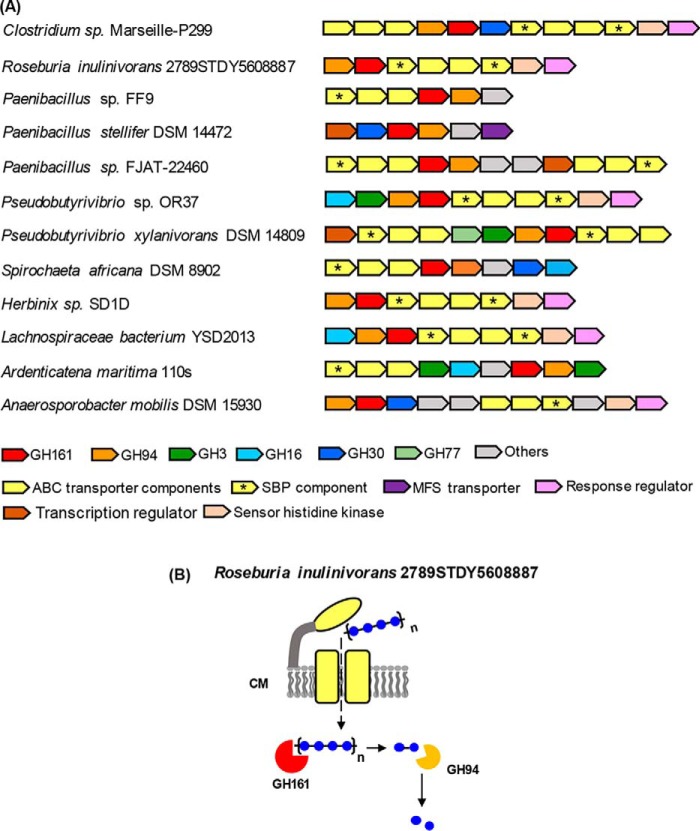
**Genetic loci containing *GH94-GH161* genes in tandem and possible roles of the enzymes encoded by the genetic loci.** *A*, gene clusters containing putative *GH94-GH161* genes in tandem arrangement. *B*, possible roles of GH94 and GH161 in sequential degradation of oligosaccharide substrate. ABC transporter is *colored* in *yellow* and located in the cell membrane (*CM*). The subcellular localization prediction of GHs and transporters was performed by PSORTb version 3.0.2 (http://psort.org/psortb/index.html)^5^ using default settings for Gram-positive bacteria. SBP components of the ABC transport system are indicated by *asterisks*.

To assess a possible physiological role for GH161 enzymes in bacteria, the locations of *GH161* genes within the corresponding bacterial genomes were inspected (Figs. 7 and 8). *PapP* gene was found to co-localize with genes encoding β-glucosidase (GH1) and an ABC transporter (Fig. 7*A*). The subcellular localization of PapP and GH1 proteins was predicted to be in the cytoplasm, suggesting a role for the PapP enzyme in degradation of oligosaccharides to disaccharides, which are then subsequently broken down to Glc by GH1 hydrolase (Fig. 7*B*). This genetic organization suggests that the *PapP* gene cluster might be involved in degradation of a simple linear oligosaccharide substrate.

A gene encoding GH161 from *Bacillus weihaiensis* Alg07 (WP_072580822.1), an alga-associated marine bacterium, maps to a previously identified laminarin utilization locus (Fig. 7*A*), which contains GH30, a β-glucosidase (GH1), and a laminarinase (GH16), all of which are highly expressed upon bacterial exposure to laminarin (38). The location of *GH161* in the laminarin utilization locus raises the possibility that the GH161 enzyme may be involved in laminarin degradation in this species. The GH30 family contains enzymes with β-(1→6)-*endo*-glucanase activity, required for degradation of β-(1→6)-linkages in β-glucans. The *B. weihaiensis* GH30 protein was predicted to be secreted by the cell, and therefore the enzyme is likely involved in the removal of β-(1→6)-linked branches on laminarin prior to the import of linear β-(1→3)-linked oligosaccharides (Fig. 7*C*). In contrast, the *B. weihaiensis* GH1 and GH161 proteins were predicted to localize to the cytoplasm; there, they are likely to be involved in sequential depolymerization of linear β-(1→3)-linked oligosaccharides into Glc (Fig. 7*C*).

Analysis of genomes from other bacterial species containing *GH161* genes revealed two distinctive patterns of gene clusters. 1) *GH161* genes co-localize with other GHs, in particular with genes encoding β-glucosidases from the GH1, GH3, and GH30 families (Fig. 7*A*). Again, this organization suggests a role for the GH161 enzymes in the depolymerization of oligosaccharide substrate, similar to that described for the *PapP* gene cluster. For example, a gene cluster from *Acholeplasma laidlawii* PG-8A contains genes encoding putative GH161, two permease components of ABC transporter, a laminarinase (GH16), and a β-glucosidase (GH3) (Fig. 7*A*, *red-dotted box*). The substrate-binding protein (SBP) component of the ABC transporter, which is important for substrate specificity and high affinity of substrate uptake (39), is missing from the *A. laidlawii GH161* gene cluster. Interestingly, a gene cluster containing a gene encoding the previously characterized *A. laidlawii* GH94 LBP (15), was found located next to the putative GH161 gene cluster (Fig. 7*A*, *black-dotted box*). The *A. laidlawii* GH94 cluster also contains genes encoding ABC transporter components, including SBP. The close proximity of the *A. laidlawii* GH161 and GH94 gene clusters and the lack of an SBP gene in the former imply that the two clusters may share the SBP component for substrate uptake and that the two clusters encode enzymes that work cooperatively in degrading the same β-glucan substrate. The same organization of GH94 and GH161 gene clusters was also observed in *Acholeplasma oculi* 19L (Fig. 7*A*). 2) Co-localization of putative GH161 with GH94 genes in a tandem manner (Fig. 8*A*) illustrates the first example of two different GP families co-existing in this pattern. As the activities observed for PapP suggest that GH161 enzymes are only capable of digesting oligosaccharides into disaccharides while GPs in the GH94 family act on disaccharides (40–42), it is likely that the GH161 and GH94 enzymes encoded by the tandem genes may act sequentially on the same oligosaccharide substrate (Fig. 8*B*).

## Discussion

Comparative analyses of protein sequences of enzymes in the GH94, GH149, and GH161 families reveal the conservation of amino acids involved in catalysis and recognition of sugar donor substrate and P_i_. This suggests the potential divergent evolution of these families from a common ancestor, while maintaining the catalytic apparatus. However, the characterization of representatives of each family indicates different substrate preferences; GH94 LBP only phosphorolysed G2, whereas the characterized GH149 and GH161 are oligosaccharide-acting enzymes, operating on longer substrates. Among GH149 and GH161, the minimum chain lengths required for phosphorolysis are different: GH149 required at least DP 2 (Fig. 1, *n* ≥ 0), whereas GH161 required at least DP 3 (Fig. 1, *n* ≥ 1). The difference between the substrate chain length preferences in GH94, GH149, and GH161 indicates divergence in the structure of the acceptor substrate-binding subsites between these enzymes. It is interesting to note that the tolerance toward β-linked disaccharide acceptors with different linkage regioselectivity observed in both Pro_7066 and PapP indicates a somewhat conserved recognition of the acceptor substrate linkage stereochemistry, but not regiochemistry, among GH149 and GH161 enzymes (Fig. 5 and Figs. S3, S4, and S5).

The majority of identified GPs are predicted to be involved in carbohydrate degradation, largely due to the co-localization of the corresponding genes with those encoding sugar transporters and other GHs (14, 24, 43, 44). We previously mapped the location of genes encoding bacterial GH149 enzymes to clusters containing sugar transporters and other GH family genes. In particular, the Bacteroidetes *GH149* genes were found to locate to previously identified polysaccharide utilization loci (PULs) (45, 46), implicated in the degradation of β-(1→3)-glucans (24). The importance of PULs containing GH149 in laminarin degradation was reinforced by a recent comprehensive study of the PUL from *Formosa* sp. Hel1_33_13 and its role in laminarin turnover during diatom-dominated spring blooms (47). Expression of the *Formosa* PUL was detected in metagenome and metaproteome analyses of water samples collected during the bloom, suggesting a correlation between the up-regulation of PUL expression and the utilization of laminarin by the bacterium. In addition, recombinant expression and characterization of a GH30 and two GH17 enzymes in the *Formosa* PUL strongly implicated these enzymes in laminarin degradation (47). Based on the notion that GH161 enzyme may have a role in glycan degradation, the lack of phosphorolytic activity on disaccharides in GH161 enzymes has an important physiological implication: the disaccharide product could not be used in downstream carbon metabolic processes unless the same organism encodes additional GHs or GPs that can degrade them to free Glc and Glc1P. This is supported by the observed co-localization of genes encoding GH161 with those encoding β-glucosidases and predicted GH94 enzymes, which presumably degrade disaccharide products of GH161 catalysis to produce Glc.

We have observed the co-localization of *GH94* and *GH161* genes in some species during the gene cluster analysis, providing the first examples of co-localization of two GP-encoding genes from different families. A similar tandem arrangement of GP-encoding genes has also been observed in a gene cluster containing two GH130 β-(1→2)-mannoside phosphorylases from *Thermoanaerobacter* sp. X-513, which was proposed to be involved in degradation of β-(1→2)-mannoside to supply α-d-mannose 1-phosphate for GDP-d-mannose biosynthesis (48). Based on this notion, it is possible that the purpose of gene clusters containing the *GH94-GH161* tandem is to degrade β-glucan from an external source to supply Glc1P for other metabolic pathways, such as storage glycan synthesis and glycolysis. The occurrence of *GH94-GH161* tandem genes points toward genetic co-inheritance of the two families, the enzyme activities of which have evolved to operate on glucan substrates with different lengths (disaccharide for GH94 *versus* oligosaccharide for GH161) in a functionally coordinated manner to ensure complete oligosaccharide feedstock degradation.

ABC transporters were found in most of the GH161-containing genetic loci analyzed in this study. Surprisingly, the majority of the genes lack nucleotide-binding domains (NBDs), which are required for ATP binding, the hydrolysis of which typically provides the energy needed to drive the conformational changes that enable substrate uptake (49). It is possible that incomplete ABC transporters found in *GH161* gene clusters may share the NBD component with other ABC transport systems, which is a reasonable assumption, considering the fact that NBD components are highly conserved in all ABC transporter systems (49, 50). A similar hypothesis has been proposed for a recently identified ABC transporter for β-(1→2)-glucan uptake in *Listeria innocua*, in which the gene encoding a putative NBD was found in a distant location in the genome and did not cluster with the genes encoding the remaining ABC transporter components (51).

GH161 candidates were identified predominantly in *Paenibacillus* spp., suggesting an important role of GH161 in this genus. Comparison between GH161-containing gene clusters from *Paenibacillus* spp. showed that different *Paenibacillus* species adopt different genetic organizations of the *GH161* gene clusters. However, the inheritance of the entire gene cluster seems to be conserved among different strains of the same species (*i.e. P. polymyxa* ATCC842, E681, and CR1 all share the same *GH161*-containing gene cluster) (Fig. 7*A*).

In conclusion, the identification of a new GH family, GH161, and characterization of a GH161 member, PapP, extend the repertoire of known GPs acting on β-(1→3)-d-glucans, albeit with different acceptor substrate preference from GH94 and GH149. Analysis of gene clusters containing *GH161* genes provides the first evidence of probable coordination between GH94 and GH161 enzymes in sequential degradation of oligosaccharide substrates. Further characterization of other co-localized GHs and the transporters encoded in the GH161-containing gene clusters could provide further support to the significance of these gene clusters and their encoded proteins in oligosaccharide degradation.

## Experimental procedures

### Recombinant PapP protein expression and purification

*PapP* gene sequence was amplified from *P. polymyxa* ATCC 842 genome using the following primers: forward primer, 5′-AAGTTCTGTTTCAGGGCCCGATGCAGCCGTATTA-3′; reverse primer, 5′-ATGGTCTAGAAAGCTTTATTAAAGCGATCTCTCCAG-3′. The amplified sequence was ligated into pOPINF plasmid vector (36) following the manufacturer's protocol. The recombinant *pOPINF-PapP* was transformed into Stella competent cells for plasmid propagation. For protein expression, the recombinant plasmid was transformed into chemically competent *E. coli* (Rosetta PLysS) cells, and a 1-liter culture of the transformant was grown at 18 °C in LB medium containing 100 μg/ml carbenicillin with agitation (180 rpm) overnight. Heterologous protein expression was induced by adding isopropyl 1-thio-β-d-galactopyranoside to a final concentration of 0.2 mm and incubating for 1 day at 18 °C.

The *E. coli* cells expressing PapP were harvested (6,721 × *g*, 10 min) and lysed by sonication in buffer A (10 mm HEPES, pH 7.5, 250 mm NaCl) supplemented with 1 mg/ml DNase (Sigma) and 1 tablet of complete protease inhibitor mixture (Roche Applied Science). Supernatant containing the recombinant proteins was separated from cell debris by centrifugation (32,914 × *g*, 30 min). Proteins were purified with the ÄKTA pure FPLC system (GE Healthcare) at 4 °C. The supernatant containing His_6_-tagged recombinant protein was loaded to a 1-ml HisTrap^TM^ HP column (GE Healthcare) pre-equilibrated with buffer A. The column was washed with buffer A, and bound proteins were eluted in one step with buffer B (10 mm HEPES, pH 7.5, 250 mm NaCl, 500 mm imidazole). The proteins were further purified by gel filtration using a Superdex S200 16/600 column (GE Healthcare), eluted with 20 mm HEPES, pH 7.5, 150 mm NaCl, 1 ml/min. Fractions containing the proteins were pooled and concentrated to 10 mg/ml using Amicon Ultra-15 30,000 molecular weight cut-off concentrator. The proteins were stored in 30-μl aliquots at −80 °C until required.

### Recombinant TaCDP protein expression and purification

*TaCDP* gene sequence was synthesized and optimized for *E. coli* expression by Eurofins Genomics. The sequence was amplified by PCR and ligated into pOPINF plasmid vector (36) following the manufacturer's protocol. The recombinant *pOPINF-TaCDP* was transformed into Stella competent cells for plasmid propagation. For protein expression, the recombinant plasmid was transformed into chemically competent *E. coli* BL21 (DE3) cells, and a 1-liter culture of the transformant was grown at 37 °C in LB medium containing 100 μg/ml carbenicillin with agitation (180 rpm) overnight. Heterologous protein expression was induced by adding isopropyl 1-thio-β-d-galactopyranoside to a final concentration of 1 mm and incubating for 1 day at 18 °C. IMAC purification of the TaCDP protein was performed in the same manner as described previously for PapP.

### Enzymatic assays

The phosphorolysis of G2 and G3 were carried out in 20 μl of an assay buffer (20 mm oligosaccharides, 10 mm KH_2_PO_4_ in 100 mm HEPES, pH 7.0 (all concentrations are final concentrations) and 1 μl of recombinant protein (Pro_7066 or PapP, 10 mg/ml stock solution). The reaction mixture was incubated for 1 h at 30 °C. The reaction was stopped by boiling (5 min), and oligosaccharide products were analyzed by TLC analysis, HPAEC-PAD, and MALDI-TOF.

The glycan synthetic reactions were carried out in 20 μl of an assay buffer (100 mm HEPES, pH 7.0, 10 mm Glc1P, 10 mm Glc-Glc disaccharide acceptors, incubated with 1 μl of recombinant protein (Pro_7066 or PapP, 10 mg/ml stock solution) at 30 °C for 1 h. The reaction was stopped by boiling (5 min), and oligosaccharide products were analyzed by TLC analysis or HPAEC-PAD and MALDI-TOF.

Kinetic parameters of PapP were determined using reaction mixtures (20 μl) containing the enzyme (25 μg/ml) in the presence of 0.2–10 mm G2, G3, laminaritetraose (G4), laminaripentaose (G5), or G6, 10 mm Glc1P, and 200 mm sodium molybdate in 100 mm HEPES, pH 7.0, buffer (all concentrations are final concentrations). The amount of phosphate released was measured by a phosphate release assay (52) with the following modification. The enzymatic reaction was stopped by boiling (5 min) and left to cool to room temperature. A color solution (90 μl, 13.6 mm sodium ascorbate in 0.1 m HCl) was added to the boiled reaction mixture and incubated for 30 min at room temperature to allow color development. A stop solution (90 μl, 68 mm sodium citrate tribasic dihydrate in 2% acetic acid) was added to the mixture to stop the color development. The absorbance of final solution was measured at 620 nm on a 96-well plate reader. The amount of phosphate released was calculated from the absorbance by comparing with a phosphate standard curve ranging between 0 and 10 mm. All assays were performed in triplicates. The values of released phosphate were fitted on nonlinear regression with a Michaelis–Menten model using GraphPad Prism to determine *V*_max_ and *K_m_*.

### Oligosaccharide analyses

TLC was performed by spotting 0.5 μl of the recovered reaction mixture onto precoated slides of Silica Gel 60 F254 (Merck) (10 × 5 cm) and then eluted using a mobile phase containing NH_4_OH/H_2_O/isopropyl alcohol (3:1:6) in a sealed glass container for 2 h to allow oligosaccharide separation. The plate was air-dried and stained with orcinol, which was prepared by adding concentrated sulfuric acid (20 ml) to an ice-cold solution of 3,5-dihydroxytoluene (360 mg) in ethanol (150 ml) and water (10 ml). The stained plate was then heated until oligosaccharide spots were visible.

HPAEC-PAD analyses were performed by diluting the reaction mixtures in MilliQ water to a final volume of 500 μl and desalted by mixed-bed ion-exchange resin (Sigma). The desalted mixtures were filtered through a disposable PTFE 0.45-μm filter disc (Merck Millipore) and subjected to HPAEC-PAD analysis using a Dionex ICS3000 chromatography system equipped with PAD and controlled by Chromeleon® software. A PA100 CarboPac column (analytical, 4 × 250 mm; guard, 4 × 50 mm) was used for all analyses with a mobile phase composed of 100 mm sodium hydroxide (solution A) and 100 mm sodium hydroxide + 400 mm sodium acetate (solution B). The separation was achieved by gradient elution: 0–100% solution B over 30 min, followed by 20 min of 100% B and then a 10-min re-equilibration of the column with 100% solution A. The solutions were delivered to the column at a rate of 0.25 ml/min.

### Bioinformatic analysis and phylogeny

Orthologous sequences to OcP1 were obtained from the nonredundant protein sequence database and the MMET Sequencing Project, using BLASTP or tBLASTn with an *E*-value score of 0.0001 or better. Multiple-sequence alignments of amino acid sequences were performed using Clustal Omega (53) (www.clustal.org/omega,^5^ version 1.2.2) with the default settings and edited with trimAl version 1.2 using a heuristic automated method (54). The alignments were visualized by Jalview (version 14.6.4) (55). Phylogenetic trees were reconstructed from a matrix of 306 unambiguously aligned amino acids using PhyML version 3.0 (56) with the best fit model as inferred by a smart model selection (SMS). Bootstrap values were determined from a population of 100 replicates. Tree annotation and visualization were performed using iTOL version 3.4.3 (57). The GenBank^TM^ numbers of the GH161 members can be found in File S1.

## Author contributions

S. K., N. J. P., and R. A. F. conceptualization; S. K. data curation; S. K., G. P., and B. H. formal analysis; S. K., N. J. P., B. H., and R. A. F. validation; S. K. and G. P. investigation; S. K. visualization; S. K., G. P., N. J. P., B. H., and R. A. F. methodology; S. K. writing-original draft; S. K., N. J. P., B. H., and R. A. F. writing-review and editing; N. J. P. and R. A. F. supervision; R. A. F. resources; R. A. F. funding acquisition; R. A. F. project administration.

## Supplementary Material

Supporting Information

## Footnotes

4 S. Kuhaudomlarp, C. E. M. Stevenson, D. M. Lawson, R. A. Field, submitted for publication.

5 Please note that the JBC is not responsible for the long-term archiving and maintenance of this site or any other third party hosted site.

## Footnotes

3 The abbreviations used are:

GP: glycoside phosphorylase
GH: glycoside hydrolase
MMET: Marine Microbial Eukaryote Transcriptome
DP: degree of polymerization
ABC: ATP-binding cassette
LBP: laminaribiose phosphorylase
IMAC: immobilized metal affinity chromatography
SBP: substrate-binding protein
PUL: polysaccharide utilization locus
LB: lysogeny broth
HPAEC: high-performance anion-exchange chromatography
PAD: pulsed amperometric detection
TLC: thin layer chromatography
G2: G3, G4, G5, G6, etc., laminaribiose, laminaritriose, laminaritetraose, laminaripentaose, laminarihexaose, etc.
