# Supplementary material for: Unraveling the subtleties of β-(1→3)-glucan phosphorylase specificity in the GH94, GH149, and GH161 glycoside hydrolase families
Source: J Biol Chem. 2019 Feb 28;294(16):6483–93. doi: 10.1074/jbc.RA119.007712 (PMC6484121; doi:10.1074/jbc.RA119.007712)
Supplement: Supporting Information [file supp_294_16_6483__index.html]

Unraveling the subtleties of β-(1→3)-glucan phosphorylase specificity in the GH94, GH149 and GH161 glycoside hydrolase families — New enzyme family containing β-(1→3)-glucan phosphorylases — Unraveling the subtleties of β-(1→3)-glucan phosphorylase specificity in the GH94, GH149, and GH161 glycoside hydrolase families — New enzyme family containing β-(1→3)-glucan phosphorylases — Supporting Information 

# Unraveling the subtleties of β-(1→3)-glucan phosphorylase specificity in the GH94, GH149, and GH161 glycoside hydrolase families

## Supporting Information

- Supporting Information (to be published online) - Supporting Information Figures
- Supporting Information (to be published online) - GH161 sequence ID
- Supporting Information (to be published online) - BLAST analysis
