# Supplementary material for: Unraveling the subtleties of β-(1→3)-glucan phosphorylase specificity in the GH94, GH149, and GH161 glycoside hydrolase families
Source: J Biol Chem. 2019 Feb 28;294(16):6483–93. doi: 10.1074/jbc.RA119.007712 (PMC6484121; doi:10.1074/jbc.RA119.007712)
Supplement: Supporting Information [file supp_RA119.007712_143342_1_supp_287288_pnjbj2.pdf]

Supporting Information

Unraveling the subtleties of β-(1→3)-glucan phosphorylase specificity in the GH94, GH149, and GH161 glycoside hydrolase families

Sakonwan Kuhaudomlarp,[a,b] Giulia Pergolizzi,[a] Nicola J. Patron,[c] Bernard Henrissat,[d,e,f] Robert A. Field[a,1]

[a]Department of Biological Chemistry, John Innes Centre, Norwich Research Park, Norwich NR4 7UH, UK. [b] Present address: Univ. Grenoble Alpes, CNRS, CERMAV, 38000 Grenoble, France. [c]Earlham Institute, Norwich Research Park, Norwich, NR4 7UZ, UK. [d]Aix-Marseille University, Architecture et Fonction des Macromolécules Biologiques, 163 Avenue de Luminy, 13288 Marseille, France. [e]Centre National de la Recherche Scientifique, UMR 7257, 163 Avenue de Luminy, 13288 Marseille, France. [f]Department of Biological Sciences, King Abdulaziz University, Jeddah, Saudi Arabia.

Running title: new enzyme family containing β-1,3-glucan phosphorylases

[1]To whom correspondence should be addressed: Robert A. Field: Department of Biological Chemistry, John Innes Centre, Norwich Research Park, Norwich NR4 7UH, UK. rob.field@jic.ac.uk.

Keywords: heterokont, β-1,3-glucan, Gram-positive bacteria, orthologs, carbohydrate utilization loci, carbohydrate metabolism, glycoside hydrolase, phosphorylase, glycobiology, enzyme evolution

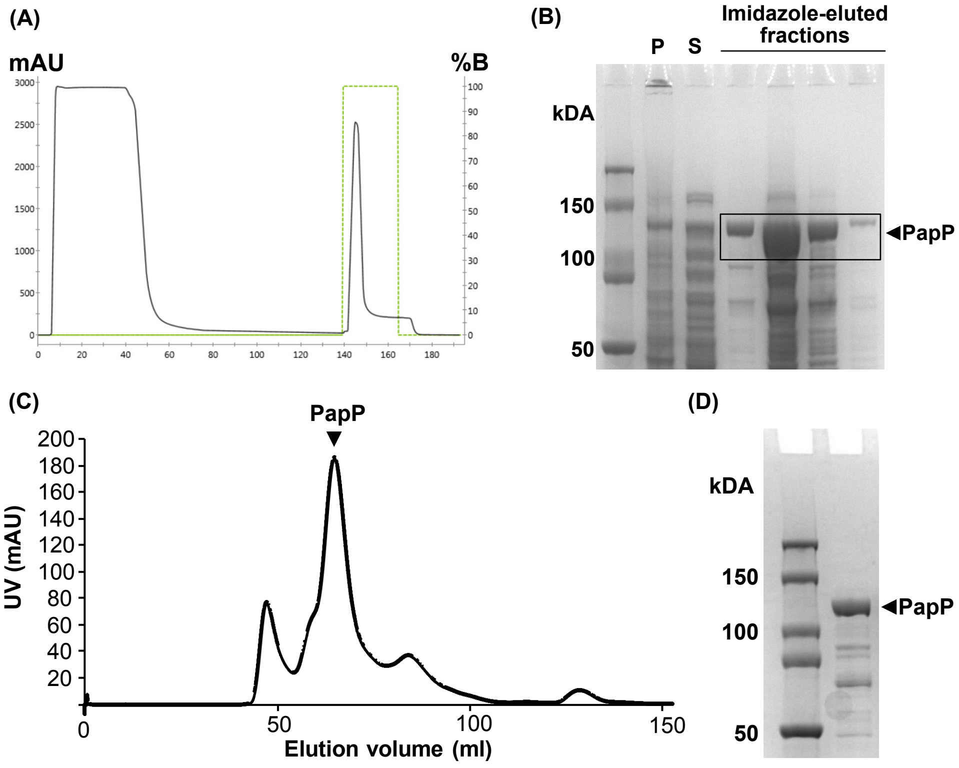

Figure S1. Production of recombinant PapP protein in *E. coli* and purification of the soluble protein. (**A**) IMAC purification with UV detection trace (black line). PapP was eluted in 100% of buffer B (10 mM HEPES pH 7.5, 250 mM NaCl, 500 mM imidazole) (green line). (**B**) SDS-PAGE of PapP after IMAC purification. P = pellet, S = supernatant. (**C**) Gel filtration of PapP. The protein was eluted at 65-ml elution volume in 20 mM HEPES pH 7.5, 150 mM NaCl. (**D**) SDS-PAGE analysis of the purified recombinant PapP after IMAC and gel filtration.

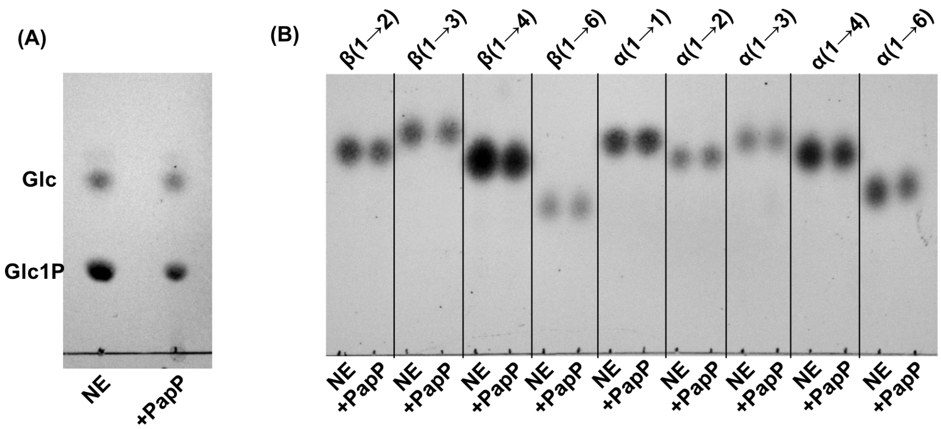

Figure S2. TLC analysis of PapP activity on Glc and Glc1P in the glycan synthesis and on disaccharides as the phosphorolytic substrates. (**A**) Glycan synthetic reaction in the presence of Glc (10 mM) and Glc1P (20 mM) as substrates. (**B**) Phosphorolysis reactions using Glc-Glc disaccharides with varying glycosidic linkages (20 mM) (as indicated at the top) as substrate in the presence of inorganic phosphate (10 mM). NE = no enzyme control.

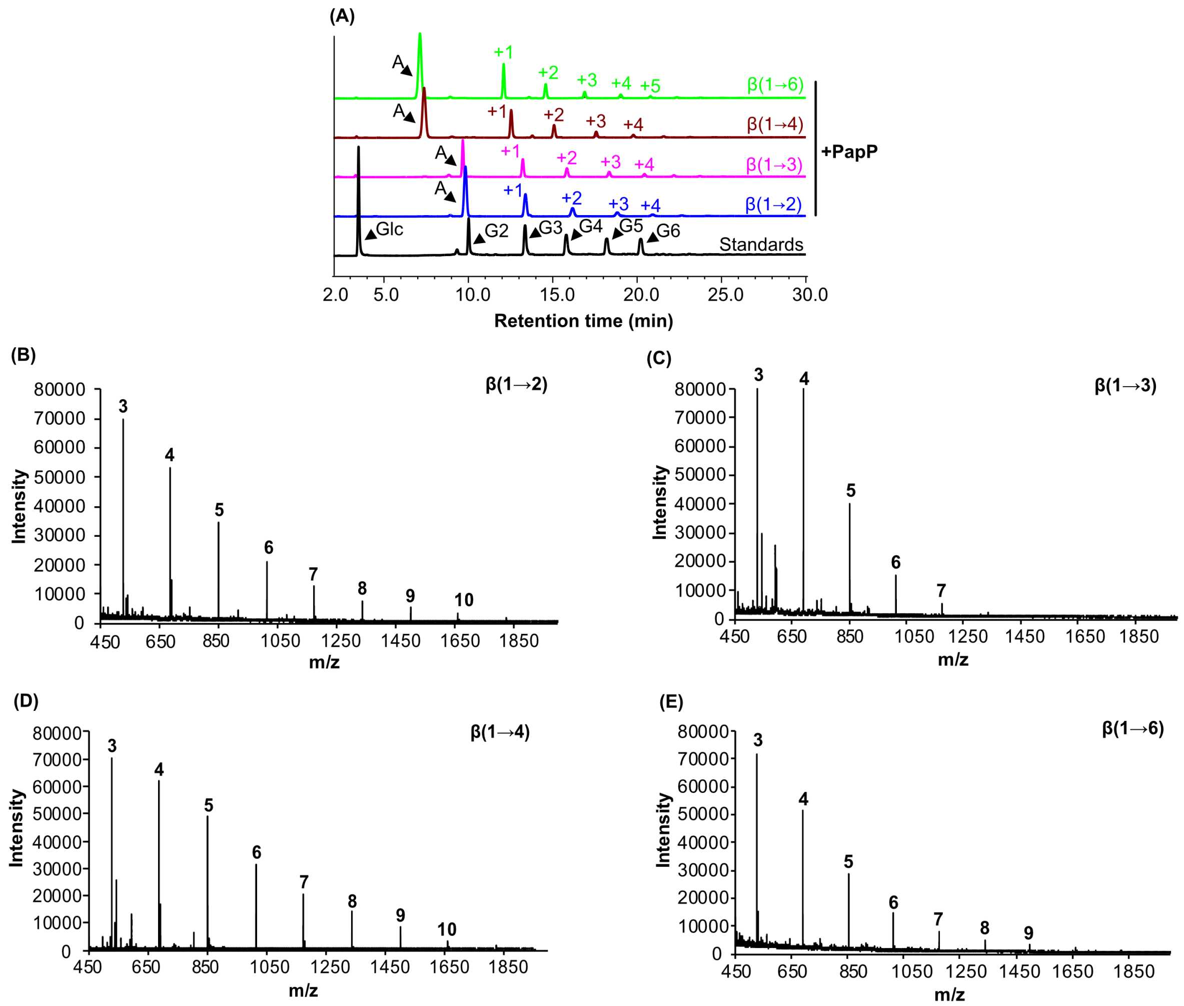

Figure S3. Glycan synthetic reactions carried out by PapP in the presence of β-linked disaccharide acceptors and Glc1P (**A**) HPAEC-PAD analysis of the glycan synthetic reaction in the presence of β-linked glucose-glucose disaccharide. A = peaks that represents the acceptors in each reaction. The linkages of the acceptors are indicated on the right. +1 to +5 = the number of Glc that have been transferred from Glc1P donor onto the acceptors, thus creating oligomers that are represented by the peaks. For example, peak labelled with +1 represents an acceptor that has been extended by 1 Glc unit, therefore has the total DP of 3. G2 = laminaribiose, G3 = laminaritriose, G4 = laminaritetraose, G5 = laminaripentaose, G6 = laminarihexaose. (**B-E**) MALDI-ToF analyses of the glycan synthetic reactions catalyzed by PapP in the presence of β-linked disaccharide acceptors and Glc1P**.** The glycosidic linkages of the acceptors are indicated in the figures. The DP of glucan products corresponding to the peaks are indicated by numbers (4-11).

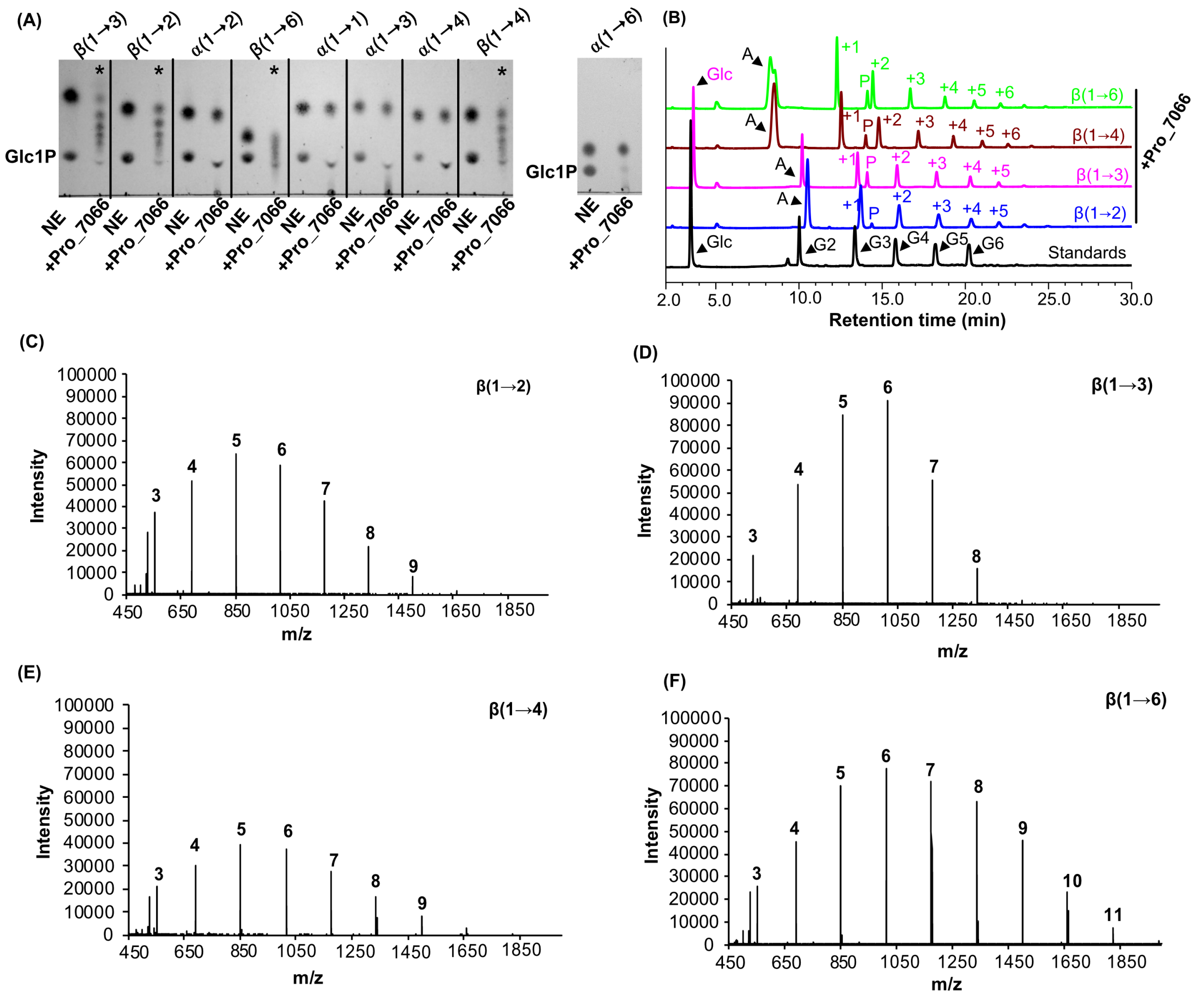

Figure S4. Glycan synthetic reactions carried out by Pro_7066 from GH149 family in the presence of β-linked disaccharide acceptors (10 mM) and Glc1P (10mM). (**A**) TLC analysis of the glycan synthesis in the presence of glucose-glucose disaccharides (linkages as indicated above the figure). Asterisks indicate the detectable extension of the acceptors carried out by Pro_7066. Reaction containing α-(1→6) disaccharide as an acceptor was analyzed on a separate TLC plate. Interestingly, we observed weak spots in the Pro_7066-catalysed reactions containing α-linked acceptor substrates, which were confirmed as β-(1→3)-gluco-oligosaccharides through *exo*-β-(1→3)-glucanase treatment in Fig. S5. (**B**) HPAEC-PAD analysis of the glycan synthetic reaction in the presence of β-linked Glc-Glc disaccharide. A = peaks that represent the acceptors in each reaction. The linkages of the acceptors are indicated on the right. +1 to +5 = the number of Glc that have been transferred from Glc1P donor onto the acceptors, thus creating oligomers that are represented by the peaks. For example, peak labelled with +1 represent an acceptor that has been extended by 1 Glc, therefore has the total DP of 3. P = trace amount of Glc1P that was not completely removed before HPAEC-PAD analysis. G2 = laminaribiose, G3 = laminaritriose, G4 = laminaritetraose, G5 = laminaripentaose, G6 = laminarihexaose. (**C**-**F**) MALDI-ToF analysis of glycan synthesis using Pro_7066 as a catalyst and with β-linked disaccharides and Glc1P as substrates. Glycosidic linkages in the disaccharide acceptors are indicated. The DP of glucan products corresponding to the peaks are indicated by numbers (4-11).

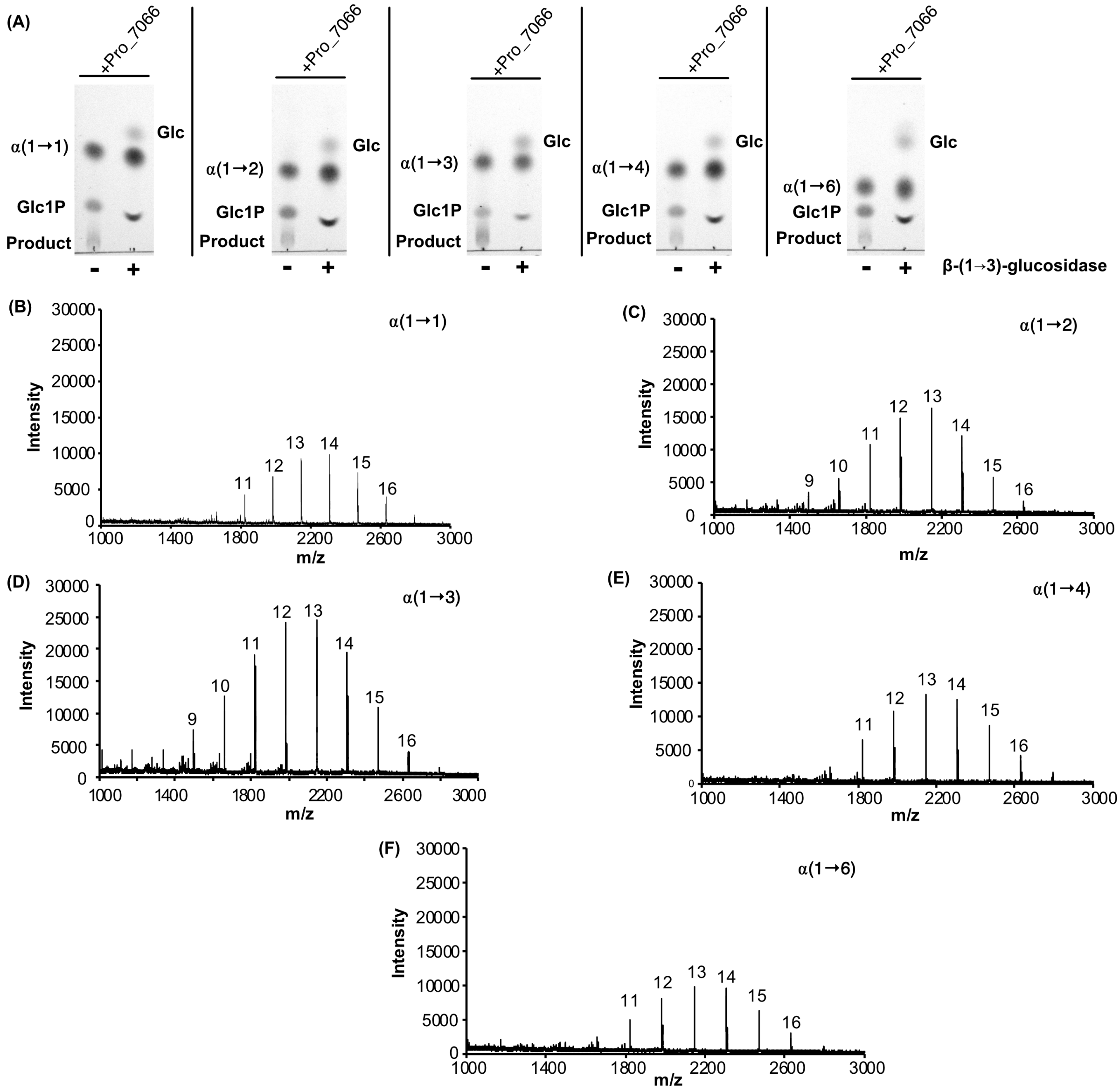

Figure S5. *exo*-β-(1→3)-Glucanase treatment of glycan products from the Pro_7066-catalyzed glycan synthetic reactions containing α-linked acceptors and Glc1P substrates. **(A)** TLC analysis of the glycan synthetic reactions before (-) and after (+) *exo*-β-(1→3)-glucanase treatment. (**B**-**F**) MALDI-ToF analyses of the glycan synthetic reactions before *exo*-β-(1→3)-glucanase treatment. The linkages of the acceptor substrates are indicated in each figure. MALDI-ToF analysis indicate the products with DP 9-16, which is longer than what we expected for the Pro_7066-catalyzed reaction in 1 hr incubation time.

Table S1. Intron analysis of the *GH161* genes from heterokonts of which their genomes have been reported on GenBank.

Species and strain	RefSeq ID	Nucleotide ID	Gene loci	Introns
Phaeodactylum tricornutum CCAP 1055/1	XP_002177898.1	NC_011670.1	PHATRDRAFT_ 43370	4
Phaeodactylum tricornutum CCAP 1055/1	XP_002181175.1	NC_011680.1	PHATRDRAFT_ 54686	1
Thalassiosira pseudonana CCMP1335	XP_002290006.1	NC_012067.1	THAPSDRAFT_ 22483	4
Thalassiosira pseudonana CCMP1335	XP_002297139.1	NW_002243509.1	THAPSDRAFT_ 25518	9
Thalassiosira oceanica CCMP1005	EJK69967.1	AGNL01009288.1	THAOC_08721	6
Aureococcus anophagefferens	XP_009032155.1	NW_008705561.1	AURANDRAFT_ 10068	2
Symbiodinium microadriaticum	OLP88051.1	LSRX01000830.1	AK812_ SmicGene30670	2
Fragilariopsis cylindrus CCMP1102	OEU10145.1	KV784373.1	FRACYDRAFT_ 194280	1
Fragilariopsis cylindrus CCMP1102	OEU15658.1	KV784359.1	FRACYDRAFT_ 170346	2

Table S2. Summary of the estimated molecular masses of PsLBP, Pro_7066, EgP1 and PapP based on their elution volumes from gel filtration column (Superdex S200 16/600, flow rate: 1 ml/min).

Proteins	Elution volume (ml)	Estimated molecular mass (kDa)	Calculated mass of monomer (kDa)
**PsLBP**	65	240	102
**Pro_7066**	62	323	130
**EgP1**	63	286	127
**PapP**	65	241	124

Table S3. Summary of the observed $[M+Na]^+$ of glucans in MALDI-ToF analysis in Fig. 4C, Fig. S3, S4 and S5.

DP of glucan	Calculated $[M+Na]^+$	The closest observed $[M+Na]^+$
**4**	689.221	689.215
**5**	851.274	851.291
**6**	1013.326	1013.345
**7**	1175.379	1175.367
**8**	1337.432	1337.422
**9**	1499.485	1499.464
**10**	1661.538	1661.499
**11**	1824.581	1823.563
**12**	1986.723	1985.589
**13**	2148.865	2148.414
**14**	2311.008	2310.156
**15**	2473.150	2469.867
**16**	2635.293	2632.539

OcP1 sequence from MMET database

CAMPEP_0173155066 /NCGR_PEP_ID=MMETSP1105-20130129|13870_1
/TAXON_ID=2985 /ORGANISM="Ochromonas sp., Strain BG-1" /LENGTH=1229
/DNA_ID=CAMNT_0014071393 /DNA_START=1 /DNA_END=3689
/DNA_ORIENTATION=-

XNRTSNNEYQAIPGDETNLELPSWNNDINSRDSALSSQNVKNPSHSKRLSLIGVSVILF
GIFAYGTYLAFSASLQPPLLADTNSEFDAQNRYILRDYDLTRPMSNFLNGLATVWGIP
LWAFYVNRGQAITSFGKQNKDAAIAKFVTAEKAYFQTPFTGFRTFLKGKRNGDSWN
HMPFFPTSEEKRAKLQRNMMIGLNELEIEEVSSEHQLQTNILYFVAPGQDFPSLVRRT
TFTNLDSYNSLELEVLDGLAQLVPNGLGNSAIDNMGRTMEAWMNVYNVGFTQGTD
TKITQPFFHISQGTADSAQVQIVRDGFFSLAYVEKTSDKASTKANAIDSENLHDLLPF
VVDPSVVFSTDTTLTNPSGFFDFSGSVEELTQSSQGTTSRTPCSFAGVRVTIPPGANVT
VTSVYGYAESLETLVGKYSPIVRXVKYSRDKRQLAYDFVADITKRVDTKTSSDVFDA
YVKQDFLDNFLRGGLPLLLGGKTTAGNSVTSPSSKVFHVYSRIHGDIERDYNYFQIDT
TYFSQGPGNFRDVCQNRRVDVSHSPFVGDFNIRLFLSFIQSDAFNPLTIASTVFKVPNT
QLEFVLDSLKILNPDGSLTNNGVGSGGLHREAARTLLSKAFRPGQFFKDAATAGVSF
AISKEEVADIIIGFAVQDFAGQFSQNGYWSDHWTYILDLVDNYLTVFPDKEAQLLWD
SEPVAFYVSPAVVKPRHSRYVAMDNPAKPGSSVLRVYNAISVWGDSQFPVEKTNAM
NAIFQDPNYLVDVNGAGSVWQRSVKDNSVVRVSVIAKLLLLGIVKFSTLDPYGLGV
EMEGGKPGWNDAMNGLPGLLGSGMPETYEMLRILRYTHSALLKFSRPVSFPSEFAD
FLTQLAAAIDRYNSSPKQLADEFVYWDSANTARETYRATVVATFKGEFKSLAASDIV
VLLEKFIAKVEGGIQRALAVNSNNGFLSPTYFYYECSQGNYEISTDGVQTSIIAKSFEL
RTLPLFLEGPTRHLRVVQTVEERRSIYEKVKSSALYDSALKMYTLCESLAAMGQEVG
RMKAFSPGWLENQSVWLHMSYKFYLELLRGGLYEEFFSEMATGLVPFMDNKVYGR
SPLEAASFIVSSAFPDKKLHGASFLARLSGSTAEFLSMWLLIVSGHQPFSVDPQTKELL
LSLQPILPGNFFDEDDEVSFVFLGKVDVVYHNPSREDTWKISAKRATVTKLDGSVVE
ASDAVIRGDVALLVRNLQAKRVDVYF
